# Recurrence of pigmented villonodular synovitis of the knee: A case report with review of literature on the risk factors causing recurrence

**DOI:** 10.1097/MD.0000000000019856

**Published:** 2020-04-17

**Authors:** Yushun Fang, Qingsong Zhang

**Affiliations:** Department of Orthopaedic Surgery, Wuhan Fourth Hospital, Wuhan City, China.

**Keywords:** arthroscopic, knee, pigmented villonodular synovitis, recurrence, tenosynovial giant cell tumor

## Abstract

**Rationale:**

Pigmented villonodular synovitis is a rare disease which may involve any joints. It has localized and diffuse forms, and the latter is more aggressive with a higher recurrence rate. Different treatments are applied to each form of the disease, but there is no standard surgical procedure or any consensus on whether adjuvant therapy should be used. Many factors may lead to recurrence of the disease; however, there is no reliable way to predict the recurrence.

**Patient concerns:**

A 21-year-old female patient presented with a one-year history of progressive pain of the right knee.

**Diagnoses:**

Pigmented villonodular synovitis.

**Interventions:**

We performed an anterior approach arthroscopic synovectomy and a posterior approach open synovectomy in the popliteal fossa, but the patient declined to take radiotherapy as a post-surgical adjuvant therapy. Then, she received a repeat arthroscopic synovectomy 20 months later because of the recurrent lesions, and a radiotherapy was performed 6 weeks after the second surgery.

**Outcomes:**

There were no abnormal signs in the right knee on magnetic resonance imaging scanning 6 months after the second surgery. The range of motion of her right knee was normal.

**Lessons:**

Pigmented villonodular synovitis is a rare disease which may involve any joints. Surgical resection plus adjuvant therapy is recommended for patients with risk factors of recurrence.

## 1 Introduction

Pigmented villonodular synovitis (PVNS) and tenosynovial giant cell tumor are considered to be one disease because of identical histological and genetic features.^[1]^ Although it has been debated for many years regarding the inflammatory and neoplastic features of PVNS,^[2–7]^ West et al^[8]^ proposed that tenosynovial giant cell tumor and the more aggressive PVNS are essentially the same disease comprised of mono-nuclear and multi-nuclear cells. However, Mrinal et al^[9]^ still attribute it to the locally aggressive connective tissue tumors, a family of lesions that usually involve the joint synovia, bursae, tendon sheath, and fibrous tissue adjacent to the tendon.^[10]^ PVNS presents as localized and diffuse forms based on the growth pattern and clinical behavior, the latter is more aggressive. While any location is possible, the localized forms mainly involve the digits and wrist, whereas the diffuse forms mainly involve large joints such as knee, hip, ankle, and elbow.^[11]^ Histopathological examination is accepted as the gold standard for the final diagnosis of PVNS.

The standard treatment for PVNS is surgical excision.^[12,13]^ Arthroscopic synovectomy and open synovectomy are the most widely used approaches. A small number of cases were treated with total knee replacement.^[14,15]^ Adjuvant therapy may be considered for patients who have a high risk of recurrence such as with diffuse PVNS.^[16,17]^ Nevertheless, the disease still has a certain rate of recurrence.

Here we report a case of recurrent diffuse intra-articular and extra-articular PVNS in an adult and we review the published literature to identify possible risk factors for recurrence of PVNS.

## 2 Case report

**Figure 1:**
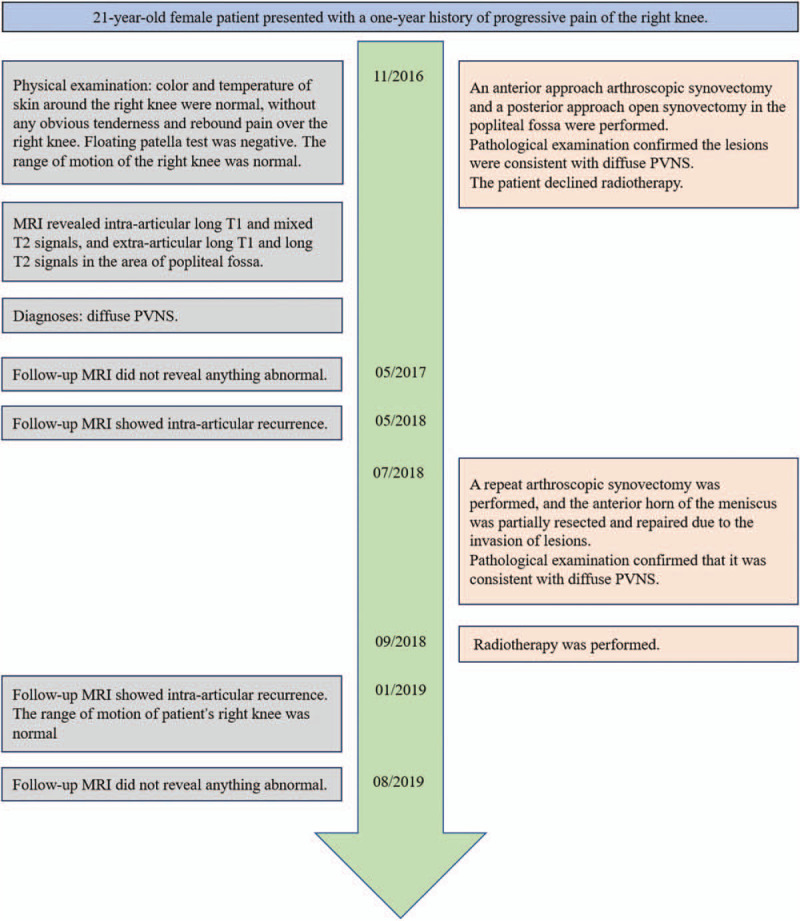
Timeline.

**Figure 2:**
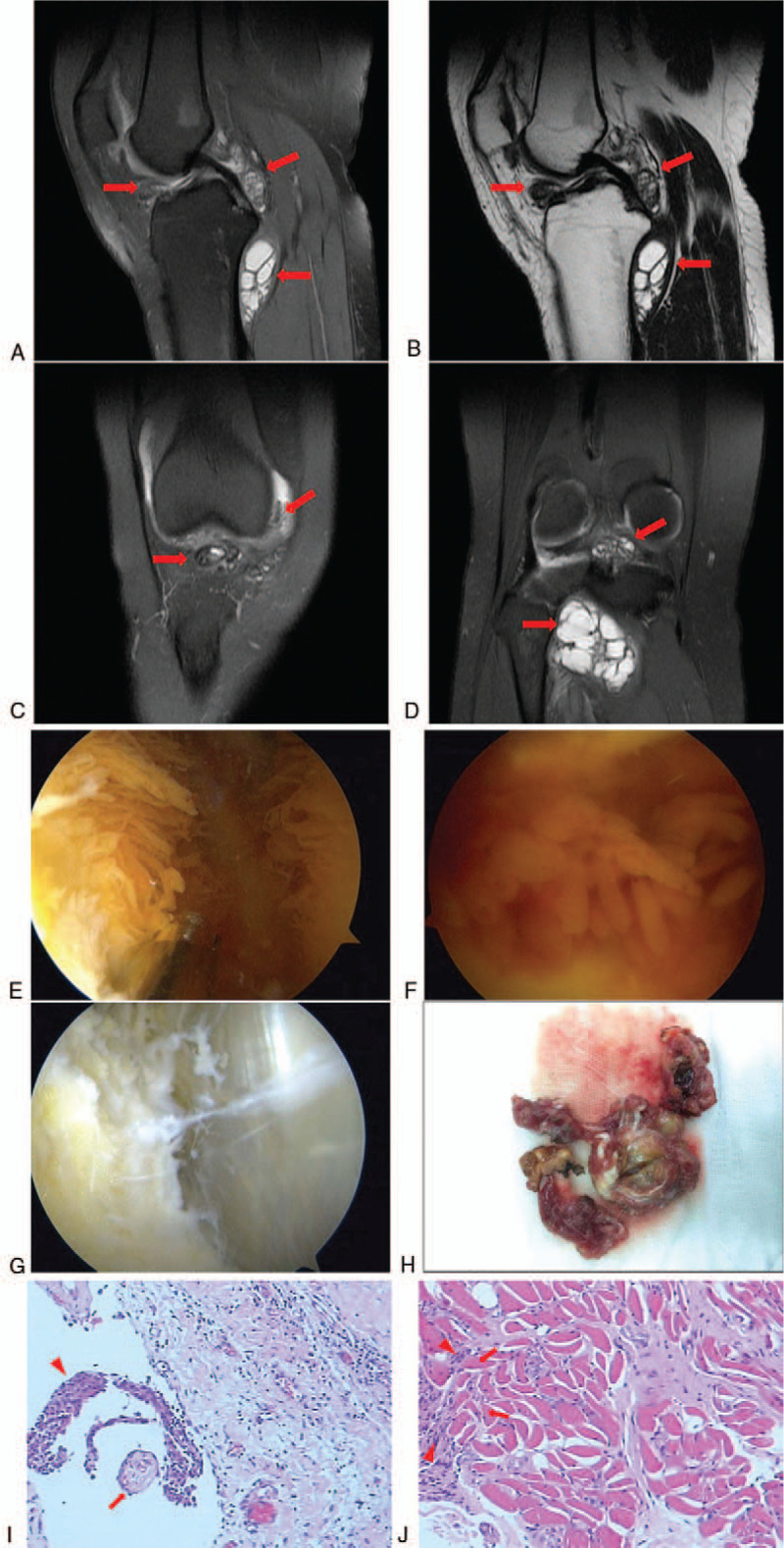
Magnetic resonance imaging of the right knee before the first surgery. (A) Sagittal MRI T2WI sequence and (B) sagittal MRI T1WI sequence shows the intra-articular and extra-articular lesions (arrows). (C, D) Coronal MRI T2WI sequence shows the intra-articular and extra-articular lesions (arrows). Synovectomy with arthroscopic anterior approach combined with open posterior approach was performed in in the initial surgery. (E, F) Intraoperative arthroscopic pictures demonstrating synovial proliferation suggestive of pigmented villonodular synovitis. (G) Intraoperative arthroscopic pictures demonstrating the intra-articular lesion had been completely resected. (H) The extra-articular lesion excised. Pathological examination of the initially excised tissues after hematoxylin and eosin staining. (I) The excised synovial tissue presented with hypertrophied synovium (arrows) and histiocytes (arrowheads), typical of pigmented villonodular synovitis. (J) Excised extra-articular tissue showed popliteus muscle (arrows) with invasion of hyperplastic histiocytes (arrowheads). Magnification, ×200. MRI = magnetic resonance imaging.

A 21-year old female patient who began to suffer from right knee pain 1 year ago referred to clinic in November 2016 in that she was being subject to deterioration condition in recent 2 months without any treatment (Fig. 1). She denied any history of trauma, previous illness, or any history of familial genetic disease, except seafood allergy. On physical examination, color and temperature of skin around the right knee were normal, without any obvious tenderness and rebound pain over the right knee. Floating patella test was negative. The range of motion of the right knee was normal. Both of blood C-reactive protein level and erythrocyte sedimentation rate were normal. The number of white blood cells was 9.5 × 10^9^/L, neutrophil count was 5.27 × 10^9^/L, lymphocyte count was 3.18 × 10^9^/L, and the neutrophil-lymphocyte ratio was 1.66. Magnetic resonance imaging (MRI) revealed intra-articular long T1 and mixed T2 signals, and extra-articular long T1 and long T2 signals in the area of popliteal fossa (Fig. 2A–D). Intra-articular synovial lesions and extra-articular popliteal lesions were diagnosed based on her disease history, laboratory and image examination.

We performed an anterior approach arthroscopic synovectomy and a posterior approach open synovectomy in the popliteal fossa in November 2016. During the surgery, we saw diffuse hyperplasia of the yellow-brown synovium in the entire right knee joint (Fig. 2E and F). Lesions in suprapatellar bursa, posterior compartment of the knee, and infrapatellar fat pad were particularly remarkable. In the area of popliteal fossa, we saw the yellow-brown nodular tissues invading into the popliteus muscle. All visibly obvious lesions were completely removed (Fig. 2G), including all synovium and fat pad as well as portion of the popliteus muscle involved (Fig. 2H). Pathological examination confirmed that the intra-articular and extra-articular lesions were consistent with diffuse PVNS (Fig. 2I and J). Due to the diffuse nature of PVNS with a high risk of recurrence, the patient was recommended to take radiotherapy as a post-surgical adjuvant therapy; however, the patient declined because of the concerns about the side effects of radiotherapy.

**Figure 3:**
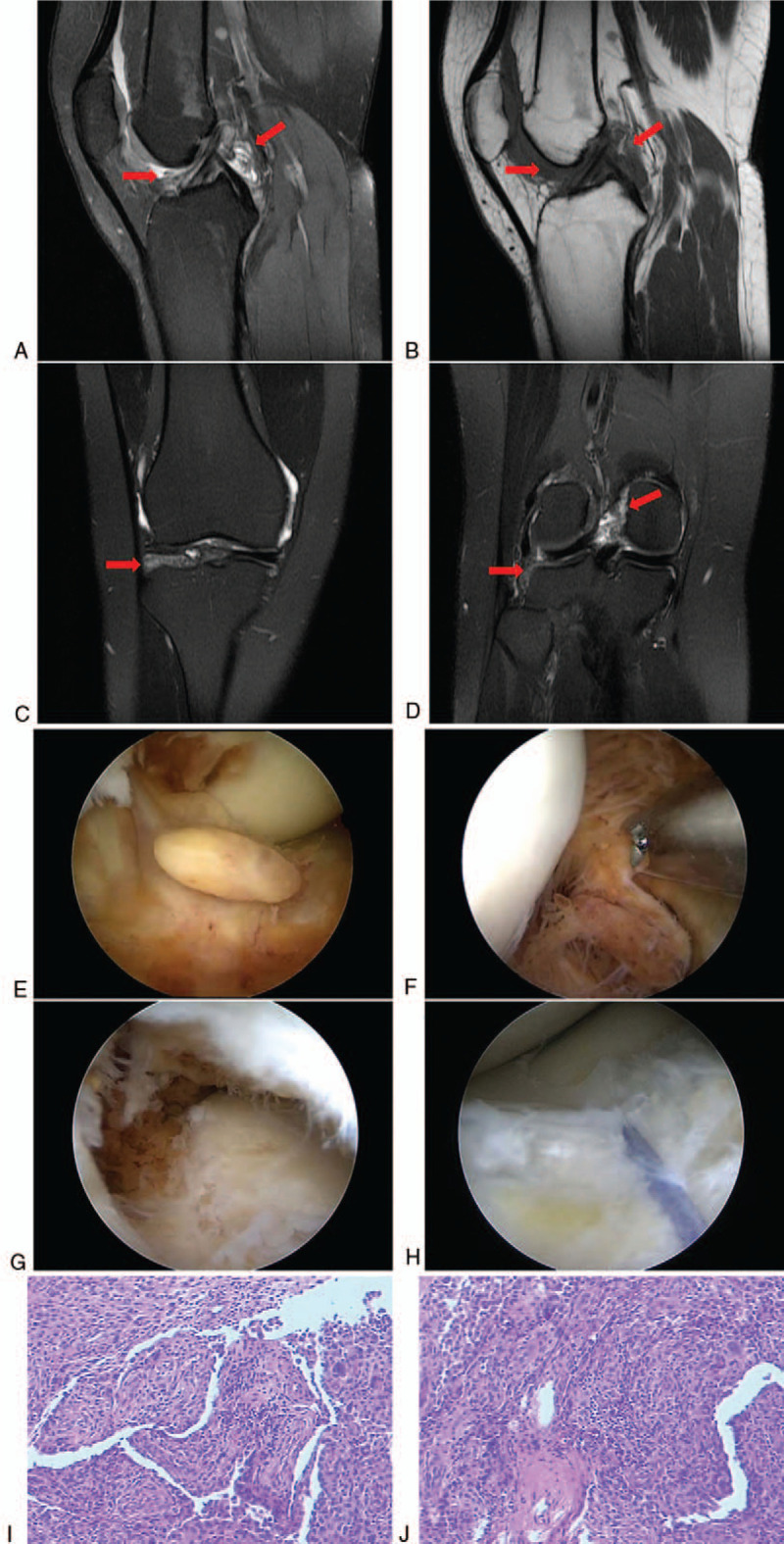
Follow-up MRI performed 18 mo after the initial surgery. (A) Sagittal MRI T2WI sequence and (B) sagittal MRI T1WI sequence showed the intra-articular recurrent lesions (arrows). (C, D) Coronal MRI T2WI sequence showed the intra-articular recurrent lesions (arrows). Arthroscopic synovectomy was performed in the second surgery. (E) Intraoperative arthroscopic picture demonstrating synovial proliferation suggestive of recurrent pigmented villonodular synovitis. (F) Intraoperative arthroscopic picture demonstrating the recurrent lesion in posterior compartment of the knee. (G) Intraoperative arthroscopic picture demonstrating the recurrent lesion under the meniscus. (H) Intraoperative arthroscopic picture demonstrating that the meniscus was repaired after resection of the lesion. Pathological examination of the tissues excised in the second surgery. (I, J) Hematoxylin and eosin staining revealed hypertrophic synovium typical of pigmented villonodular synovitis. MRI = magnetic resonance imaging.

**Figure 4:**
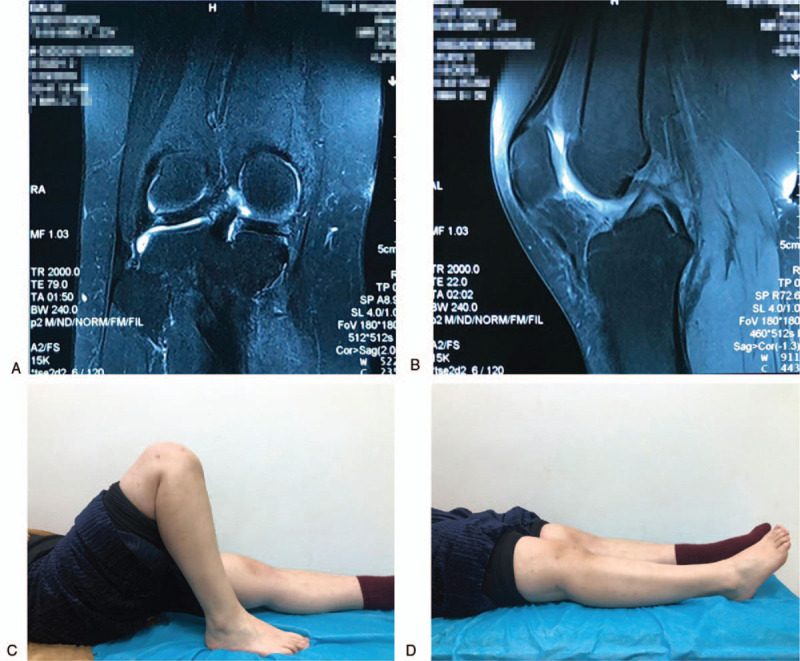
Follow-up MRI performed 6 mo after the second surgery. (A) Sagittal and (B) coronal MRI T2WI sequences showed that there was no recurrence. (Patient information for this figure has been blocked intentionally). (C, D) Postoperative assessment found that the range of motion of the right knee was normal. MRI = magnetic resonance imaging.

**Figure 5:**
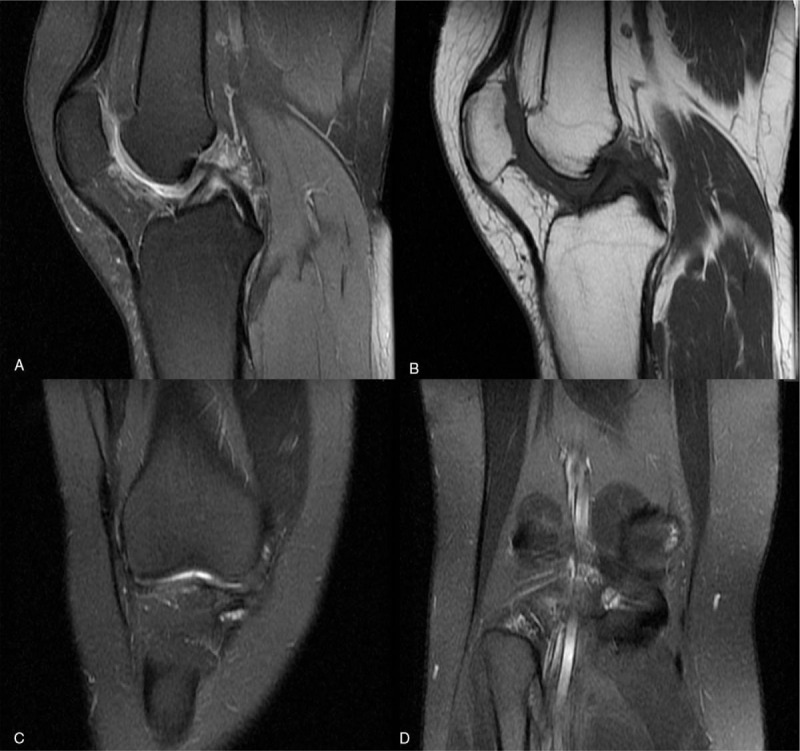
Follow-up MRI performed 13 mo after the second surgery. (A) Sagittal MRI T2WI and (B) Sagittal MRI T1WI sequences did not show recurrence. (C, D) Coronal MRI T2WI sequence did not indicate recurrence. MRI = magnetic resonance imaging.

Follow-up MRI performed 6 months after surgery did not reveal anything abnormal. However, eighteen months after the initial surgery, MRI showed multiple lesions in the suprapatellar bursa, posterior compartment of the knee, and the space under the meniscus (Fig. 3A–D). No pronounced sign of recurrence was figured out in the popliteal fossa. She received a repeat arthroscopic synovectomy in July 2018, in which all synovial lesions were confirmed (Fig. 3E–G). The lesions were resected and the anterior horn of the meniscus was partially resected and repaired (Fig. 3H). Pathological examination of the excised tissues confirmed the consistency with diffuse PVNS (Fig. 3I and J). Six weeks after the second surgery, the patient received radiotherapy. Six months after the second surgery, the patient took a follow-up MRI that did not reveal anything abnormal in the right knee (Fig. 4A and B) and the range of motion of her right knee was normal (Fig. 4C and D). Also, follow-up MRI performed again in August 2019 did not reveal anything abnormal (Fig. 5A–D).

### 2.1 Ethical statement

All procedures performed in studies involving human participants were in accordance with the ethical standards of the ethical committee in our hospital and with the Helsinki declaration and its later amendments or comparable ethical standards. Informed written consent was obtained from the patient for publication of this case report and accompanying images. This study was performed in accordance with relevant guidelines and regulations.

### 2.2 Review of literature and discussion

Diffuse form of PVNS and incomplete tumor resection are two risk factors of recurrence reported in the literature.^[18]^ However, there are no quantitative parameters to effectively predict it. Although Zhao et al^[19]^ suggested that preoperative neutrophil-lymphocyte ratio of more than 2.42 could be a valuable marker to predict the recurrence of knee PVNS, long-term follow up was not carried out to support this proposal. In our case, the ratio was less than 2.42, but the patient still had a recurrence after the first surgery. Other factors may be more important, such as the growth pattern and clinical behavior. It has been reported that localized PVNS rarely recurs, with rates of 0% to 15%.^[10,20,21]^ On the other hand, diffuse PVNS has high recurrence rates varying from 9% to 46%.^[22–27]^ Our case belonged to the diffuse form and recurred after the initial surgery. In addition, extra-articular invasion of the lesions has been reported to have a high rate of recurrence.^[28]^ Complete excision of all lesions has been recognized as the critical measure in prevention of recurrence.^[13]^ For localized PVNS, simple excision of the lesion is the gold standard of surgery, while for diffuse PVNS, adequate synovectomy is very important.^[12]^ The surgical approaches include open surgery, arthroscopic surgery, and a combination of both.^[29]^ Some reports advocated open surgery.^[23,27,30–32]^ Patel et al reviewed 214 cases and found that open surgery had statistically significant lower recurrence rate than arthroscopic surgery.^[33]^ However, Aurégan et al reported that they performed arthroscopic synovectomy combined with chemical synovectomy of any residual lesions in 7 patients with diffuse form PVNS, and none of the patients recurred during a mean follow-up of seven years.^[34]^ Keyhani et al^[35]^ treated 21 patients with diffuse PVNS by arthroscopic synovectomy and no recurrences happened during a follow-up of at least 5 years. They considered that the attentive arthroscopic synovectomy is a safer alternative with better clinical outcomes. Nevertheless, Gortzak et al thought that regarding recurrence, the comparison between open surgery and arthroscopic synovectomy was inconclusive.^[36]^ A meta-analysis of 1019 patients revealed that local recurrence after arthroscopic surgery was 16%, compared to 23% after open surgery, though there was no significant difference between the 2 approaches.^[37]^ Nevertheless, it has been widely agreed that arthroscopic surgery has better functional results and less postoperative complications than that of open surgery.^[37,38]^

A previous report found that, for the intra-articular lesions complicated with extracapsular posterior disease, anterior arthroscopic surgery combined with posterior open synovectomy was more effective than a single synovectomy.^[39]^ Mollon et al also found that a combination of arthroscopic and open surgery might allow for more thorough removal and is applicable to all tumor resection while limiting patient morbidity, especially for high-risk situations associated with both intra- and extra-articular extension.^[40]^ Based on these reports, we took the combined arthroscopic and open surgical approaches in treating our patients. However, our patient eventually demonstrated intra-articular recurrence, which may be caused by subtotal arthroscopic synovectomy as a risk factor.

Incomplete synovectomy has been associated with 44% to 55% of recurrences.^[24,41]^ A previous survey has shown that the recurrence rate was also dependent on the number of cases treated at the institutions, for example, a recurrence rate of 56% was reported in institutions with less than 20 cases per year, whereas a rate of 15% to 37% was found in institutions with more than 20 cases per year.^[42]^ One possible explanation for this is that experience and meticulous surgery is significant when applying resection to the PVNS lesions.

Many nonsurgical therapies have been tested, including tyrosine kinase inhibitors, radiofrequency ablation,^[17]^ external beam radiotherapy,^[16]^ and radiosynovectomy using intra-articular radioactive isotopes, such as Rhenium 186, Phosphorus 32, and Yttrium 90.^[22,36]^ In recent years, some people believe that conservative treatment may achieve certain curative effects, for example, systemic antibody treatment with PLX3397 (a selective colony stimulating factor 1 receptor inhibitor) has shown some favorable results.^[43]^ Imatinib mesylate is another tyrosine kinase inhibitor that has activity against colony stimulating factor 1 receptor inhibitor,^[44]^ displaying some activities against PVNS in a recent report. Lalam et al applied radiofrequency ablation to treat localized PVNS and achieved certain effects.^[17]^ Nonsurgical treatment is more often used as postoperative adjuvant therapy to reduce recurrence.

Radiotherapy is the most widely used adjuvant treatment after surgery. It has been shown that radiotherapy reduces recurrence in diffuse PVNS, especially in cases with incomplete synovectomy.^[45]^ Ottaviani et al^[22]^ recommended intra-articular radiotherapy after partial synovectomy for diffuse PVNS due to the difficulty in completely resecting of all lesions.^[22]^ Gouina et al proposed that adjuvant treatment is necessary after synovectomy for diffuse PVNS.^[11]^ Isotopic synoviorthesis or external radiotherapy may be considered after primary resection, and systemic treatment by targeted therapy or radiation therapy may be an option after synovectomy in cases of recurrence or rapid progression. After arthroscopic synovectomy and radiotherapy, few cases recurred.^[46]^ Adjuvant radiosynovectomy with Yttrium-90 has been used in many patients to reduce recurrence rates.^[47]^ On the other hand, Gortzak et al treated 56 PVNS patients with a mean follow-up of 7.3 years and found that there were no significant differences in the outcomes between the patients treated surgically with or without an adjuvant intra-articular injection of 90 Yttrium.^[36]^ Mazonakis et al found that knee irradiation may result in an inconsequential risk for carcinogenesis irrespective.^[48]^ Another study also found that skin cancer is clearly a potential risk, thus caution is advised when considering radiotherapy for these conditions in children and young adults.^[49]^ A report showed that external-beam irradiation for PVNS might have a role in the development of two aggressive neoplasms at the primary treatment site.^[50]^ These concerns over the side effects of radiotherapy hindered the application of adjuvant radiotherapy after the initial therapy. However, after her lesions recurred, she decided to take adjuvant radiotherapy after the second surgery and her disease has not recurred so far.

In addition, delayed diagnosis is a crucial risk factor for recurrence and poor prognosis,^[51]^ which could be rescued by MRI^[29]^ and postoperative follow-up. Research has shown that two-thirds of local recurrence were diagnosed during the first 2 years and <10% after 3 years, and follow-up MRI every 6 months during the first 3 years might be able to detect >90% of all local recurrences.^[38]^ Therefore, in order to detect recurrence in time, regular and timely follow-up is required.

For recurrence PVNS of the knee, surgical resection, either open or arthroscopic, is considered the main strategy of treatment.^[52]^ Jobe et al considerate that it is necessary to excise some otherwise normal-appearing fat and areolar tissue with the synovium in recurrences cases.^[53]^ And external beam radiation is an option as adjuvant therapy.^[38]^ There were no severe complications reported in most studies on recurrence PVNS.

## 3 Conclusions

PVNS is a rare disease which may involve any joints. Standard treatment is surgical resection through open surgery, arthroscopic surgery, or a combination of both. The risk factors for recurrence include diffuse form of the disease, incomplete resection, location of the lesions, the experience and skills of the surgeon, and adjuvant therapy after surgery. Surgical resection plus adjuvant therapy is recommended for patients with risk factors of recurrence.

## Author contributions

**Conceptualization:** Yushun Fang.

**Data curation:** Yushun Fang.

**Formal analysis:** Yushun Fang.

**Methodology:** Qingsong Zhang.

**Project administration:** Qingsong Zhang.

**Supervision:** Qingsong Zhang.

**Validation:** Qingsong Zhang.

**Visualization:** Yushun Fang.

**Writing – original draft:** Yushun Fang.

**Writing – review and editing:** Yushun Fang.
